# Impact of inactivated COVID-19 vaccination on female ovarian reserve: a propensity score-matched retrospective cohort study

**DOI:** 10.3389/fimmu.2023.1198051

**Published:** 2023-08-11

**Authors:** Jialyu Huang, Tianshu Guan, Lifeng Tian, Leizhen Xia, Dingfei Xu, Xingwu Wu, Lingling Huang, Mengyi Chen, Zheng Fang, Chaoyi Xiong, Liju Nie, Shuang Wang, Zengming Li, Yan Zhao, Qiongfang Wu

**Affiliations:** ^1^ Center for Reproductive Medicine, Jiangxi Maternal and Child Health Hospital, Jiangxi Branch of National Clinical Research Center for Obstetrics and Gynecology, Nanchang Medical College, Nanchang, China; ^2^ Department of Clinical Medicine, School of Queen Mary, Nanchang University, Nanchang, China; ^3^ Center for Reproductive Medicine, Department of Obstetrics and Gynecology, Tangdu Hospital, Air Force Medical University, Xi’an, China; ^4^ Department of Pathology, Jiangxi Maternal and Child Health Hospital, Jiangxi Branch of National Clinical Research Center for Obstetrics and Gynecology, Nanchang Medical College, Nanchang, China; ^5^ Department of Obstetrics and Gynecology, Jiangxi Maternal and Child Health Hospital, Jiangxi Branch of National Clinical Research Center for Obstetrics and Gynecology, Nanchang Medical College, Nanchang, China; ^6^ Key Laboratory of Women’s Reproductive Health of Jiangxi Province, Jiangxi Maternal and Child Health Hospital, Jiangxi Branch of National Clinical Research Center for Obstetrics and Gynecology, Nanchang Medical College, Nanchang, China

**Keywords:** COVID-19, SARS-CoV-2, vaccine, anti-Müllerian hormone, ovarian reserve

## Abstract

**Purpose:**

To explore the impact of inactivated COVID-19 vaccination on ovarian reserve as assessed by serum anti-Müllerian hormone (AMH) concentration.

**Methods:**

A total of 3160 women were included in this single-center retrospective cohort study between June 2021 and October 2022. Vaccination information were collected from official immunization records available in personal mobile apps. Serum AMH was qualified by electrochemiluminescence immunoassay and compared with previous measurement data within three years. Women were categorized to the vaccinated group if they received two doses of inactivated COVID-19 vaccines (Sinopharm or Sinovac) between AMH tests (*n* = 488), and to the control group if not vaccinated (*n* = 2672). Propensity score matching and multivariate linear regression were performed to control for potential confounders. The main outcome measures were the numeric AMH change and percentage AMH change between the two tests.

**Results:**

There were 474 women left in each group after matching all baseline characteristics. The mean interval from the first to second AMH measurement was 508.0 ± 250.2 and 507.5 ± 253.6 days for vaccinated and unvaccinated women, respectively (*P* = 0.680). Both groups had a significant AMH decrease in the second test compared with the first test (*P* = 0.001). However, the second AMH level remained comparable between groups (3.26 ± 2.80 *vs.* 3.24 ± 2.61 ng/mL, *P* = 0.757). Similarly, no significant differences were observed in numerical (-0.14 ± 1.32 *vs.* -0.20 ± 1.56 ng/mL, *P* = 0.945) and percentage (2.33 ± 58.65 *vs.* 0.35 ± 48.42%, *P* = 0.777) AMH changes. The results were consistent in sub-analyses for women aged <35 and ≥35 years. There were also no significant differences when vaccinated women were divided according to the time interval after vaccination: ≤30, 31–60, 61–90, and ≥91 days.

**Conclusion:**

Our study provides the first evidence that inactivated COVID-19 vaccination has no measurable detrimental effect on ovarian reserve, regardless of female age and vaccination interval. This reassuring finding adds to the safety evidence of COVID-19 vaccine in fertility, and should be useful to promote vaccine acceptance. Multicenter prospective cohort studies are needed to validate our conclusion.

## Introduction

Since its outbreak in December 2019, coronavirus disease 19 (COVID-19) has grown into a worldwide pandemic, causing massive manpower and material losses. Until July 2023, there have been more than 760 million people who suffered the disease and about 7.0 million deaths attributed to it (1). The main targets of the virus, namely severe acute respiratory syndrome coronavirus type 2 (SARS-CoV-2), are respiratory tract and lung, where the spike protein of its capsid binds to angiotensin-converting enzyme 2 (ACE2) receptor on host cell. The receptor is then cleaved by type 2 transmembrane serine protease (TMPRSS2) to facilitate viral entry into the cell, which contributes to excessive inflammation and impairs normal respiration (2). Moreover, given the co-expression of ACE2 and TMPRSS2 on ovary, uterus and placenta, a negative effect of COVID-19 on female reproduction has also been suspected (3).

To fight against the disease, several vaccines have been developed for widespread inoculation, including messenger ribonucleic acid (mRNA) vaccines, adenovirus-vectored vaccines, inactivated-virus vaccines, and protein-based vaccines. As of December 2022, nearly 13 billion people have accepted vaccination all over the world (1). Previous investigations have revealed that COVID-19 vaccine had an overall good safety profile, with common side effects reported as soreness, fatigue and myalgia (4). However, a wide concern was arisen that vaccination may interfere with human reproduction. After authorization for emergence use, data analysis of internet search showed a nearly five-fold increase in online queries regarding COVID-19 vaccine-related infertility (5). According to a survey of reproductive-aged women, only 33.5% were willing to accept the vaccine. Even with recommendations from their doctors, the proportion was still as low as 49.5% (6).

There have been accumulating studies which explored the reproductive safety of female COVID-19 vaccination. With a focus on assisted reproductive treatment cycles, most cohorts demonstrated similar outcomes in oocyte quality and embryonic competence between vaccinated and unvaccinated women (7–12). In terms of ovarian reserve, the potential effect was also assessed by comparison of serum anti-Müllerian hormone (AMH) level (13–16). However, these limited studies of relatively small sample size were mainly related to mRNA vaccine (13–16), while inactivated vaccine has not been evaluated as the most widely used COVID-19 vaccine type in China. This insufficiency indicates more clinical studies to provide real-world evidence.

The aim of our study was to investigate the effect of inactivated COVID-19 vaccination on circulating AMH concentration for assessment of ovarian reserve.

## Materials and methods

### Study design and population

This retrospective cohort study was conducted at the Center for Reproductive Medicine with ISO 9001:2015 quality control, Jiangxi Maternal and Child Health Hospital. The study was approved by the Reproductive Medicine Ethics Committee of Jiangxi Maternal and Child Health Hospital (No. 2022-09), and conducted in line with the Declaration of Helsinki. Written informed contents were provided by all patients for anonymous data use in scientific research.

Women aged 20–45 years who underwent two serum AMH tests were screened for eligibility. The second AMH data was collected between June 2021 and October 2022, and the time interval to the first AMH data was limited to three years. Vaccination information, including vaccine type, dose, and inoculation date, were obtained from screened women and confirmed in official immunization records available in personal mobile apps such as Alipay, Wechat and GanFuTong. Women were categorized to the vaccinated group if they had received two doses of inactivated COVID-19 vaccines (Sinopharm or Sinovac) between AMH measurements, and to the control group if not vaccinated. Assisted reproduction techniques were performed in all enrolled women, including intrauterine insemination and/or *in vitro* fertilization-embryo transfer. The exclusion criteria were as follows: 1) self-reported history of COVID-19 or current infection detected by SARS-CoV-2 RNA in nasopharyngeal swabs; 2) incomplete vaccination of one dose or booster vaccination of three doses; 3) administration of other vaccine types, such as adenovirus-vectored vaccine (CanSino) and protein-based vaccine (Zifivax); 4) surgical procedures performed between the two AMH tests, such as salpingectomy, ovarian cystectomy, and unilateral oophorectomy; and 5) missing information in the electronic medical records.

### Serum AMH measurement

Blood samples were centrifuged and serums were collected for AMH measurement by trained technicians in central laboratory without freezing. The level of AMH was quantified by electrochemiluminescence immunoassay using Elecsys^®^ AMH Plus on a Cobas e 801 analyzer (Roche Diagnostics, Switzerland). The assay’s measuring range was 0.01–23 ng/mL with the limit of blank as 0.007 ng/mL and limit of quantitation as 0.03 ng/mL. The analytical coefficients of variation (CV) of quality controls were 1.0–1.8% for repeatability and 2.7–4.4% for intermediate precision.

### Outcome measures

The main outcome measures were the numeric AMH change and percentage AMH change between the two tests. The numeric change was calculated as the second AMH level minus the first AMH level. The percentage change was defined as numeric change divided by the first AMH level.

### Statistical analysis

SAS version 9.4 (SAS Institute, USA) was employed for all statistical analyses. Continuous variables were presented as means ± standard deviations or medians with interquartile ranges and examined for normality by Shapiro-Wilk test, while categorical variables were summarized as numbers with percentages. For the comparison between vaccinated and control women, data were analyzed by unpaired t-test, Wilcoxon rank-sum test, or Pearson χ^2^ test as appropriate. For the comparison between first and second AMH levels from the same women, data were analyzed by paired t-test or Wilcoxon signed-rank test.

We used propensity score matching (PSM) to balance baseline parameters of the vaccinated group with those of the control group. Nearest-neighbor matching without replacement was performed in a 1:1 ratio using a caliper of 0.01. The following variables were selected as potential confounders for PSM, including age, body mass index, educational level, cigarette smoking, hypertension, diabetes, dyslipidemia, gravidity, parity, infertility duration, infertility diseases, first AMH level, and time interval between AMH examinations.

In subgroup analysis, vaccinated women were categorized according to the time interval between the last vaccine dose and second AMH measurement (i.e., ≤30, 31–60, 61–90, and ≥91 days), and the outcomes were compared by one-way analysis of variance or Kruskal-Wallis test. A multiple linear regression model was also applied to assess its independent association with AMH change. Adjusted β with 95% confidence interval (CI) was computed after controlling for the aforementioned confounders. All tests were two-tailed and *P <*0.05 was considered to be statistically significant.

## Results

A total of 3160 women were eligible for inclusion in the final analysis. Among them, 488 (15.4%) were inoculated with two doses of inactivated COVID-19 vaccines, and 2672 (84.6%) were unvaccinated. For vaccinated women, the average time interval between last vaccine dose and the second AMH measurement was 123.4 ± 94.4 days.

**Table 1** shows the baseline characteristics according to vaccination status of participants. Before matching, the two groups differed significantly in age, educational level, hypertension, diabetes, dyslipidemia, tubal factor infertility, and time interval between two AMH examinations. After PSM, 474 women were left in each group and all parameters were balanced with no significant differences. The mean interval from the first to second AMH measurement was 508.0 ± 250.2 and 507.5 ± 253.6 days for vaccinated and unvaccinated women, respectively.

**Table 1:** Baseline characteristics grouped by the vaccination status.

	Before matching		*P-*value	After matching		*P-*value
	Vaccinated (*n* = 488)	Control (*n* = 2672)		Vaccinated (*n* = 474)	Control (*n* = 474)	
Age (years)	30.73 ± 4.96	31.68 ± 5.29	0.001	30.83 ± 4.95	30.68 ± 5.32	0.277
Body mass index (kg/m^2^)	22.06 ± 3.26	22.02 ± 3.09	0.949	22.01 ± 3.21	22.07 ± 3.13	0.593
Educational level, *n* (%)			0.001			0.832
Middle school or less	132 (27.1)	926 (34.7)		131 (27.6)	139 (29.3)	
High school	85 (17.4)	495 (18.5)		81 (17.1)	77 (16.2)	
College or above	271 (55.5)	1251 (46.8)		262 (55.3)	258 (54.4)	
Cigarette smoking, *n* (%)	8 (1.6)	58 (2.2)	0.450	8 (1.7)	10 (2.1)	0.634
Hypertension, *n* (%)	17 (3.5)	50 (1.9)	0.023	13 (2.7)	13 (2.7)	1.000
Diabetes, *n* (%)	13 (2.7)	36 (1.4)	0.030	10 (2.1)	15 (3.2)	0.311
Dyslipidemia, *n* (%)	214 (43.9)	768 (28.7)	<0.001	200 (42.2)	196 (41.4)	0.792
Gravidity, *n* (%)			0.743			0.974
0	218 (44.7)	1172 (43.9)		209 (44.1)	208 (43.9)	
1	125 (25.6)	660 (24.7)		122 (25.7)	125 (26.4)	
≥2	145 (29.7)	840 (31.4)		143 (30.2)	141 (29.8)	
Parity, *n* (%)			0.444			0.623
0	342 (70.1)	1918 (71.8)		331 (69.8)	324 (68.4)	
≥1	146 (29.9)	754 (28.2)		143 (30.2)	150 (31.7)	
Infertility duration (years)	3.87 ± 2.84	4.12 ± 2.98	0.123	3.89 ± 2.87	3.87 ± 2.58	0.723
Infertility diseases						
Tubal factor, *n* (%)	298 (61.1)	1801 (67.4)	0.006	292 (61.6)	291 (61.4)	0.947
Male factor, *n* (%)	118 (24.2)	562 (21)	0.120	112 (23.6)	115 (24.3)	0.819
Ovulatory dysfunction, *n* (%)	75 (15.4)	441 (16.5)	0.533	72 (15.2)	85 (17.9)	0.256
Diminished ovarian reserve, *n* (%)	109 (22.3)	639 (23.9)	0.451	104 (21.9)	87 (18.4)	0.169
Endometriosis, *n* (%)	49 (10)	272 (10.2)	0.926	48 (10.1)	39 (8.2)	0.311
Uterine factor, *n* (%)	60 (12.3)	297 (11.1)	0.449	59 (12.5)	49 (10.3)	0.307
AMH at first test (ng/mL)	3.37 ± 2.60	3.49 ± 3.08	0.428	3.39 ± 2.61	3.45 ± 2.67	0.790
Time interval between AMH tests (days)	520.4 ± 257.8	372.4 ± 244.2	<0.001	508.0 ± 250.2	507.5 ± 253.6	0.680

Data are presented as mean ± standard deviation or number (percentage).

AMH, anti-Müllerian hormone.

**Figure 1:**
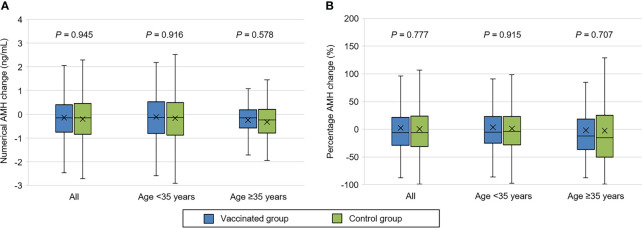
Comparison of serum anti-Müllerian hormone (AMH) change between vaccinated and control women. **(A)** Numerical AMH change. **(B)** Percentage AMH change. The symbol ‘×’ represents mean value.

As illustrated in **Supplementary Figure 1**, both groups had a significant AMH decrease in the second test compared with the first test (*P* = 0.001). However, the second AMH level remained comparable between vaccinated and control groups (3.26 ± 2.80 *vs.* 3.24 ± 2.61 ng/mL, *P* = 0.757) (**Table 2**). Similarly, no significant differences were observed in numerical (-0.14 ± 1.32 *vs.* -0.20 ± 1.56 ng/mL, *P* = 0.945) and percentage (2.33 ± 58.65 *vs.* 0.35 ± 48.42%, *P* = 0.777) change of AMH concentration. The results were also consistent in additional sub-analyses for women aged <35 and ≥35 years (**Table 2**, **Figure 1**).

**Table 2:** Serum anti-Müllerian hormone (AMH) change of vaccinated and control women.

	Vaccinated (*n* = 474)		Control (*n* = 474)		*P-*value
	Mean ± SD	Median (IQR)	Mean ± SD	Median (IQR)	
AMH at second test (ng/mL)	3.26 ± 2.80	2.65 (1.37–4.34)	3.24 ± 2.61	2.59 (1.37–4.53)	0.757
Age <35 years	3.66 ± 2.89	2.92 (1.70–4.68)	3.71 ± 2.68	3.06 (1.77–5.10)	0.432
Age ≥35 years	1.61 ± 1.54	1.15 (0.52–2.23)	1.49 ± 1.28	1.25 (0.50–2.21)	0.829
AMH change (ng/mL)	-0.14 ± 1.32	-0.14 (-0.75–0.40)	-0.20 ± 1.56	-0.14 (-0.85–0.45)	0.945
Age <35 years	-0.11 ± 1.41	-0.14 (-0.80–0.52)	-0.18 ± 1.66	-0.11 (-0.89–0.51)	0.916
Age ≥35 years	-0.25 ± 0.80	-0.15 (-0.57–0.19)	-0.29 ± 1.12	-0.24 (-0.79–0.20)	0.578
AMH change (%)	2.33 ± 58.65	-6.15 (-28.83–21.42)	0.35 ± 48.42	-5.81 (-31.53–24.15)	0.777
Age <35 years	3.38 ± 57.32	-5.00 (-25.05–22.60)	1.45 ± 44.83	-3.74 (-28.01–23.37)	0.915
Age ≥35 years	-1.98 ± 63.94	-12.53 (-36.31–18.23)	-3.81 ± 60.19	-19.03 (-51.03–25.12)	0.707

SD, standard deviation; IQR, interquartile range.

Serum AMH change in vaccinated women were further divided according to the time interval from full vaccination to second AMH test (**Table 3**). There were no significant differences in both numerical and percentage changes of AMH among the four groups: ≤30, 31–60, 61–90, and ≥91 days. Compared with the ≤30-day group, the adjusted β (95% CI) for numerical change was 0.10 (-0.31–0.52), -0.25 (-0.67–0.17), and 0.17 (-0.17–0.51), respectively. Similarly, the adjusted β (95% CI) for percentage change was 0.01 (-0.18–0.19), -0.16 (-0.35–0.03), and -0.02 (-0.18–0.13), respectively.

**Table 3:** Serum anti-Müllerian hormone (AMH) change according to different time intervals after vaccination.

	≤30 days (*n* = 68)	31–60 days (*n* = 82)	61–90 days (*n* = 73)	≥91 days (*n* = 265)	*P*-value
Interval between last vaccination and second AMH test (days)	17.5 ± 8.6	45.2 ± 8.9	76.1 ± 8.9	187.8 ± 82.5	<0.001
Age (years)	30.68 ± 5.55	31.29 ± 5.40	30.79 ± 4.92	30.56 ± 4.68	0.679
AMH at first test (ng/mL)	3.35 ± 2.68	3.02 ± 2.45	3.67 ± 2.88	3.40 ± 2.54	0.355
AMH at second test (ng/mL)	3.13 ± 2.66	3.00 ± 2.69	3.18 ± 2.62	3.32 ± 2.88	0.554
AMH change (ng/mL)	-0.22 ± 1.02	-0.02 ± 1.31	-0.49 ± 1.43	-0.08 ± 1.33	0.082
Crude β (95% CI)	–	0.20 (-0.22–0.62)	-0.28 (-0.71–0.15)	0.14 (-0.21–0.48)	
*P*-value	–	0.353	0.206	0.442	
Adjusted β (95% CI) ^a^	–	0.10 (-0.31–0.52)	-0.25 (-0.67–0.17)	0.17 (-0.17–0.51)	
*P*-value	–	0.620	0.244	0.318	
AMH change (%)	3.16 ± 47.79	10.99 ± 62.77	-12.71 ± 34.38	2.54 ± 63.55	0.055
Crude β (95% CI)	–	0.08 (-0.11–0.26)	-0.16 (-0.35–0.03)	-0.01 (-0.16–0.15)	
*P*-value	–	0.410	0.105	0.937	
Adjusted β (95% CI) ^a^	–	0.01 (-0.18–0.19)	-0.16 (-0.35–0.03)	-0.02 (-0.18–0.13)	
*P*-value	–	0.927	0.096	0.766	

CI, confidence interval.

a Analyses were adjusted for age, body mass index, educational level, cigarette smoking, hypertension, diabetes, dyslipidemia, gravidity, parity, infertility duration, infertility diseases, AMH level at first test, and time interval between AMH examinations.

## Discussion

Our retrospective cohort study revealed that inactivated COVID-19 vaccination did not accelerate age-related decline of AMH, implying no adverse impact on ovarian reserve. In addition, the comparable AMH change in different time intervals after vaccination further denied the possibility of short-term ovarian impairment.

The effect of COVID-19 vaccine on human fertility has become a subject of great concern, one of which is female ovarian reserve (17). To date, several clinical studies have made investigations on this issue with consistent results. The first prospective cohort by Mohr-Sasson et al. (13) consisted of 129 women who were inoculated with two Pfizer-BioNTech vaccines. Mean AMH levels were found to be comparable at baseline and three months post-vaccination (5.30 ± 4.29 *vs.* 5.30 ± 4.50 ng/mL, *P* = 0.11). Besides, there was no association between AMH level and the degree of immune response (as expressed by anti-SARS-CoV-2 antibody titer) after adjusting for age. Similarly, Horowitz et al. (15) prospectively enrolled 31 infertile women undergoing assisted reproductive treatment, and observed no significant change in median AMH concentrations before and after full vaccination within 4 months (1.7 *vs.* 1.6 ng/mL, *P* = 0.96). This is further confirmed in another cohort limited to young women aged 25–30 years, whose pre- and post-vaccine AMH levels were 4.17 ± 1.87 and 4.13 ± 1.94 ng/mL, respectively (*P* = 0.785) (14). In a recent study by Yang et al. (16), a direct comparison was made between new female patients with and without COVID-19 vaccination, and no significant difference was found in serum AMH after multivariate linear regression analysis (adjusted β = 0.241, 95% CI -0.054−0.536).

Notably, the aforementioned studies were all carried out on mRNA or adenovirus-vectored COVID-19 vaccine, while the impact of other vaccine types remains unclear. Their relatively small sample sizes also limit the statistical power of results. Given the wide application of inactivated vaccine in China (18), our study was conducted based on a much larger cohort involving 948 women after matching. Unlike the previous before-and-after design in vaccinated women, the AMH change, either numerical or percentage, was further compared with that in unvaccinated control women. Consistently, we demonstrated a neutral influence of inactivated COVID-19 vaccine on ovarian reserve.

AMH is a dimeric glycoprotein belonging to the transforming growth factor-β family, and plays an essential role in regulating folliculogenesis (19). Serum AMH level has been well-established to be correlated with the number of primordial follicles in female ovary. Contrary to other parameters such as antral follicle count (AFC) and follicle-stimulating hormone (FSH), it remains stable across the menstrual cycle and is thus considered as one of the best standards for ovarian reserve evaluation (20). The decrease in AMH associates with higher age, and is accelerated among older women (21). Therefore, we further performed subgroup analysis for women aged <35 and ≥35 years. The consistently non-significant results confirmed that inactivated COVID-19 vaccination did not increase the susceptibility of ovarian reserve decline in different age groups.

Vaccine-induced immunity has been suspected to be associated with possible ovarian injury. On the one hand, anti-SARS-CoV-2 antibodies are capable of passing the blood-follicle barrier and have been detected in the follicular fluid (FF) of vaccinated women (8, 22), while their biological effects remain largely unclear. Until recently, a German cohort found that there was no negative association of FF antibody titers with oocyte development and fertilization success in assisted reproduction (23). On the other hand, COVID-19 vaccine may induce autoimmune response as other vaccines (24), which could contribute to the pathogenesis of premature ovarian insufficiency (25). Indeed, a retrospective cohort study detected a significant elevation of anti-β2 glycoprotein I concentration in peripheral blood after inactivated COVID-19 vaccination, while no adverse effect was observed on *in vitro* fertilization and embryo transfer outcomes (26). Therefore, the hypothesized immunological alterations do exist, but their interference with ovarian function are denied by emerging studies as well as our cohort.

Similarly, most previous research concluded that COVID-19 vaccination did not exhibit negative effects on male fertility (7, 27–32). However, in a prior longitudinal study, Gat et al. (33) demonstrated that receipt of BNT162b2 could cause a temporary deterioration of sperm concentration and total motile sperm count followed by a recovery three months later. In the present study, the impact of time interval after vaccination on serum AMH change was also investigated, while no significant differences were found across the groups of ≤30, 31–60, 61–90, and ≥91 days. This finding is in agreement with previous studies showing that ovarian stimulation and pregnancy outcomes were not affected by different vaccination intervals of 1, 1.8, 2, 3, 3.2, 4.5, 6, or 9 months during assisted reproductive treatment (11, 34–38). It also supports the guidelines of the American Society for Reproductive Medicine as well as the European Society of Human Reproduction and Embryology that women attempting to conceive could be vaccinated at any time throughout the fertility treatment (39, 40). Taken together, our results excluded the possibility of short-term ovarian reserve decline caused by inactivated COVID-19 vaccination.

There are several limitations that should be acknowledged. Firstly, this is a retrospective cohort study with potential residual confounding and inherent bias. For instance, the history of SARS-CoV-2 infection was reported by patients without ascertainment via serum antibody measurement, which may bring about misclassification risk due to recall bias. In addition, while propensity score matching and multivariate regression analyses were performed to adjust for a variety of demographic features, other confounders that may affect ovarian function were not controlled, such as lifestyle habit, drug use, and vaccine-related side effects. Secondly, the cohort was conducted in a single reproductive center, and all included women were infertile for female or male factors. In this regard, the generalization of our finding should be confirmed in other institutes and be cautioned in fertile populations. Thirdly, despite of a high reliability, serum AMH level could still be discordant with FSH and AFC (41, 42). Therefore, a more comprehensive analysis is warranted to evaluate ovarian reserve as well as oocyte quality in future studies.

## Conclusion

In summary, our study provides the first evidence that administration of inactivated COVID-19 vaccine has no measurable detrimental effect on ovarian reserve, regardless of female age and time interval after vaccination. This reassuring finding adds to the safety evidence of COVID-19 vaccine in fertility, and should provide useful guidance for both physicians and patients to increase vaccination coverage. Multicenter prospective cohort studies are needed to validate our conclusion.

## Data availability statement

The raw data supporting the conclusions of this article will be made available by the authors, without undue reservation.

## Ethics statement

The studies involving humans were approved by Reproductive Medicine Ethics Committee of Jiangxi Maternal and Child Health Hospital. The studies were conducted in accordance with the local legislation and institutional requirements. The participants provided their written informed consent to participate in this study.

## Author contributions

ZL, YZ and QW were responsible for study conception and design. JH, LX, LT, DX, XW, LH, MC and YZ participated in data collection and entry. TG, LT and LX conducted the statistical analyses. JH and TG drafted the manuscript. ZF, CX, SW and LN were involved in data interpretation and discussion. ZL, YZ and QW supervised the project administration. All authors approved the final manuscript after critical review and revision for intellectual content.

## Supplementary material

Supplementary Figure 1 Comparison of serum anti-Müllerian hormone (AMH) concentration between two tests in **(A)** vaccinated and **(B)** control women. The symbol ‘×’ represents mean value.
