# Supplementary figures and images for: Impact of inactivated COVID-19 vaccination on female ovarian reserve: a propensity score-matched retrospective cohort study
Source: Front Immunol. 2023 Aug 11;14:1198051. doi: 10.3389/fimmu.2023.1198051 (PMC10451080; doi:10.3389/fimmu.2023.1198051)

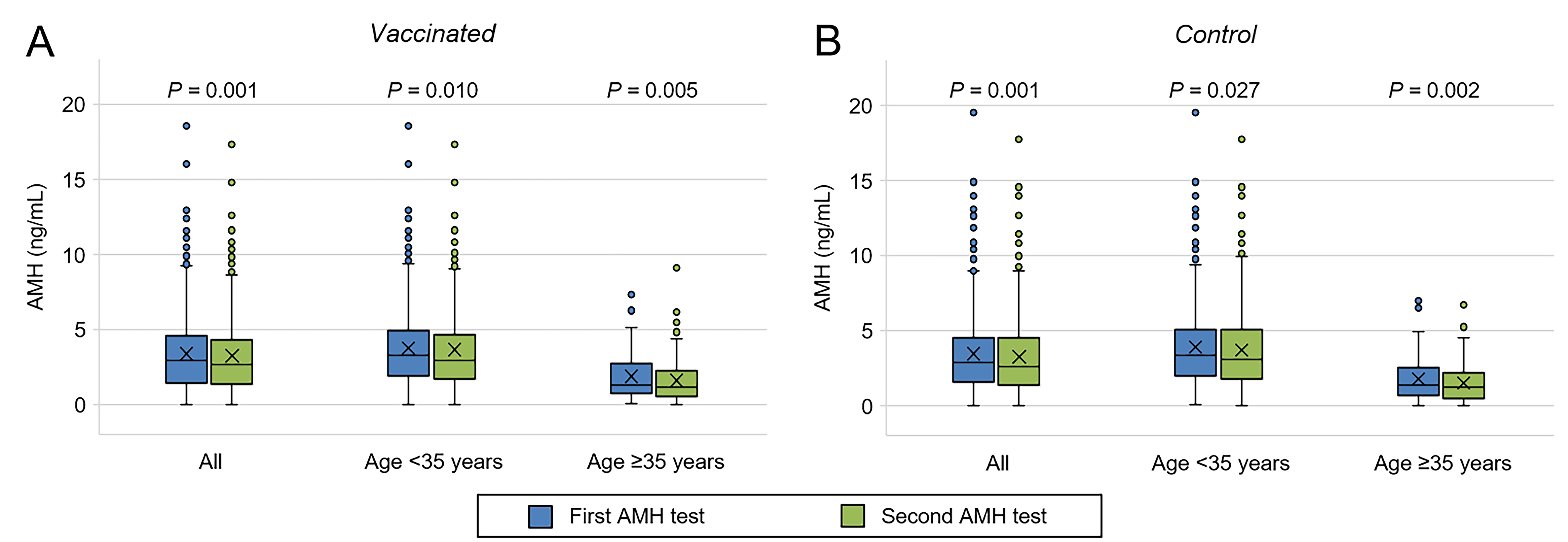

Supplement: Supplementary Figure 1 — Comparison of serum anti-Müllerian hormone (AMH) concentration between two tests in (A) vaccinated and (B) control women. The symbol ‘×’ represents mean value. [file Image_1.tif]
